# Multifaceted Roles of TRIM Proteins in Colorectal Carcinoma

**DOI:** 10.3390/ijms21207532

**Published:** 2020-10-13

**Authors:** Wolfgang Eberhardt, Kristina Haeussler, Usman Nasrullah, Josef Pfeilschifter

**Affiliations:** Institut für Allgemeine Pharmakologie und Toxikologie, Pharmazentrum Frankfurt/ZAFES, Universitätsklinikum und Goethe-Universität Frankfurt am Main, Theodor-Stern-Kai 7, D-60590 Frankfurt am Main, Germany; haeussler@med.uni-frankfurt.de (K.H.); nasrullah@em.uni-frankfurt.de (U.N.); pfeilschifter@em.uni-frankfurt.de (J.P.)

**Keywords:** apoptosis, colorectal cancer (CRC), epithelial-to-mesenchymal transition (EMT), TRIMs, oncogenic signaling

## Abstract

Colorectal cancer (CRC) is one of the most frequently diagnosed tumor in humans and one of the most common causes of cancer-related death worldwide. The pathogenesis of CRC follows a multistage process which together with somatic gene mutations is mainly attributed to the dysregulation of signaling pathways critically involved in the maintenance of homeostasis of epithelial integrity in the intestine. A growing number of studies has highlighted the critical impact of members of the tripartite motif (TRIM) protein family on most types of human malignancies including CRC. In accordance, abundant expression of many TRIM proteins has been observed in CRC tissues and is frequently correlating with poor survival of patients. Notably, some TRIM members can act as tumor suppressors depending on the context and the type of cancer which has been assessed. Mechanistically, most cancer-related TRIMs have a critical impact on cell cycle control, apoptosis, epithelial–mesenchymal transition (EMT), metastasis, and inflammation mainly through directly interfering with diverse oncogenic signaling pathways. In addition, some recent publications have emphasized the emerging role of some TRIM members to act as transcription factors and RNA-stabilizing factors thus adding a further level of complexity to the pleiotropic biological activities of TRIM proteins. The current review focuses on oncogenic signaling processes targeted by different TRIMs and their particular role in the development of CRC. A better understanding of the crosstalk of TRIMs with these signaling pathways relevant for CRC development is an important prerequisite for the validation of TRIM proteins as novel biomarkers and as potential targets of future therapies for CRC.

## 1. Introduction

Colorectal cancer (CRC) currently represents the third most prevailing cancer and a leading cause of all human cancer deaths worldwide [1,2]. Despite of a slow progression and a myriad of therapeutic approaches, CRC is characterized by high mortality, which is mainly explained by the strong metastatic potential of the primary tumor and a high recurrence [3,4]. In the majority of CRC cases (70%), the tumor appears spontaneously with a number of environmental risk factors that have been identified including adverse dietary habits, age, pathogens, and chronic intestinal inflammation with both latter ones being critically influenced by the individual microbiome [3,5,6]. A genetic basis of the cancer is only observed in approximately 30% of all CRC patients. In these cases, CRC has mainly been linked with chromosomal and microsatellite instabilities that affect the expression of oncogenes. Further genetic mechanisms underlying CRC include aberrant gene fusions, multiplications of gene copies, as well as epigenetic alterations, particularly DNA methylation and histone acetylation [7,8,9]. In addition, inherited mutations underlying familial adenomatous polyposis (FAP) or mismatch mutations due to defective DNA repair account as frequent risk factors predisposing individuals to the development of CRC [10]. Prominent examples are germline mutations in the tumor suppressor gene adenomatous polyposis coli (Apc), leading to the constitutive β-catenin mediated transcription of Wnt controlled genes critically involved in proliferation of epithelial cells. Under normal conditions, the Apc protein interacts with β-catenin together with casein kinase 1 (CK1) and glycogen synthase kinase 3 (GSK3) to form a destruction complex which renders β-catenin in an inactive state in the cytoplasm. Interestingly, mutations in the Apc gene were not exclusively found in FAP patients, but also in many sporadic colorectal tumors [11,12]. Besides the Wnt/β-catenin signaling pathway other signaling cascades critical for keeping the integrity of the intestine including the transforming growth factor β (TGFβ)-Smad signaling module, the phospho**i**nositide-3 (PI3)- and Akt kinases, the K-ras oncogene, or those driven by the tumor suppressor p53 are frequently altered in sporadic CRC [13]. In addition, a large fraction of CRC tumors exhibit a constitutive activation of prominent pro-inflammatory signaling pathways, such as signal transducer and activator of transcription 3 (STAT3) and nuclear factor κB (NF-κB) [14].

Over the last decade, a growing body of evidence has revealed that besides genetic and epigenetic events, an aberrant turnover of either oncogenic proteins or tumor suppressors by the ubiquitin proteasome system (UPS) plays a crucial role in the etiology and pathogenesis of CRC. Functionally, the UPS is established to be involved in the regulation of almost every structural and functional process inside the cell (for a review: [15]). The UPS describes a canonical chain of events triggered by different enzymes, including the E1 ubiquitin activating enzymes, the E2 conjugating enzymes, and the E3 ubiquitin ligases [16]. Ubiquitin-mediated proteolysis is initiated by the covalent attachment of multiple ubiquitin molecules to the target protein, followed by degradation by the 26S proteasome [16,17]. Currently, there are only two known E1 ubiquitin activating enzymes, while more than 40 E2 conjugating enzymes and more than 600 E3 ligases are described in the human genome [18,19]. In this scenario, E3 ligases act as scaffold proteins which facilitate the interaction between E2 conjugating enzymes and the corresponding substrate due to their high substrate recognition [20]. Consequently, E3 ligases ensure the specificity of ubiquitin-triggered modifications by mediating the transfer of ubiquitin to only selected substrate proteins. By using different combinations of E2 and E3 ligases, the UPS allows a high functional diversity. Notably, the ubiquitin system is not exclusively relevant for degradation of proteins, but is additionally involved in the regulation of protein–protein interactions and many diverse intracellular processes—e.g., receptor internalization, intracellular trafficking, regulation of signaling modules, modulation of transcription factors, and chromatin modification [21]. The activation of these distinct cellular features is achieved through diverse lysine (K)-specific ubiquitination. In addition to the proteolytic functions induced by tagging substrates via K48 or K11-linked ubiquitin chains, non-degradative processes are mediated through K63-linked ubiquitin [22]. In recent years, considerable progress has been made in elucidating the specific roles of E3 ligase-mediated ubiquitination in tumorigenesis. Accordingly, different types of really interesting new gene (RING) finger E3 ligases have been assigned to be critically involved in the development of CRC including RING finger proteins (RNFs), the murine double minute 2 (Mdm2) protein, the inhibitor of apoptotic proteins (IAPs), and the tripartite motifs (TRIMs) just to name a few of them [23]. Among the RING type E3 ligases the TRIM proteins represent the largest subfamily and the one which is most often deregulated in the cancer paradigm. Most of the TRIM members are critically involved in cellular key functions including transcription, cell growth and differentiation, apoptosis, and immune responses mainly via interfering with the prevalent cell signaling cascades mentioned above.

Members of the TRIM family are evolutionarily highly conserved proteins structurally characterized by an N-terminal RING finger domain, one or two zinc finger motifs named B-box domains (B1 and B2), an associated coiled-coil domain (CCD), followed by a highly variable C-terminal domain with the SPRY domain representing the most common variant in human TRIMs [24,25]. Until now, more than 70 TRIM proteins have been identified in humans and, according to differences in the organization of C-terminal domains, TRIMs were further categorized into 11 subfamilies (C-I to C-XI) [26,27]. Notably, some of the abovementioned fundamental cell responses are influenced independently of the E3 ligase activity, as indicated by the full functionality of corresponding RING deleted TRIM mutations. Vice versa, some TRIM members (e.g., *TRIM16* and *TRIM20*) although containing no typical RING domain, can exert ubiquitin ligase activity due to a cryptic RING-like structure within their B-box domains [28]. By contrast, the B-box and CCD are mainly involved in protein–protein interactions including homo-and/or heterodimerization with other TRIM proteins. Interestingly, the C-terminal domains of some TRIM members, in addition to showing protein–protein interactions, exert specific affinities to RNA [29], or enable TRIM assembly to chromatin as demonstrated—e.g., for *TRIM24* [30], *TRIM25* [31], and *TRIM29* [32].

Emerging evidence indicates that several TRIMs in addition to the well described role in innate immunity have vital roles in human tumorigenesis. Interestingly, opposing effects of aberrant TRIM expression on carcinogenesis can occur ranging from oncogenic to mainly tumor-suppressive effects depending on the protein targeted by the respective TRIM. While the majority of TRIM proteins have been assigned to act mainly as oncogenes due to the bad prognosis and outcome of patients with high tissue expression levels of the corresponding TRIM protein, only a few members including *TRIM3* [33], *TRIM8* [34], *TRIM15* [35], *TRIM45* [36], *TRIM58* [37], and *TRIM68* [38] can elicit tumor suppressive activities. In accordance, some of these tumor suppressive TRIM members are downregulated in CRC [39]. Hence, depending on cancer type and context, the same TRIM members may exert dual effects on oncogenesis. The underlying mechanisms of this dichotomy presumably depend on tumor-type-specific requirements as will be discussed in more detail in the next paragraphs. For an in-depth overview of the significance and therapeutic implication of the other RING-type E3 ligases in colorectal cancer we refer to a review by our colleagues [18].

## 2. Diverse Oncogenic Signaling Pathways Are Affected by TRIMs

Given the large number of TRIMs and their roles in diverse biological processes it is not surprising that alterations of TRIM expression levels are closely linked to a large variety of human malignancies including CRC. Accordingly, since the multitude of TRIMs are characteristically overexpressed in tumors, TRIMs are increasingly recognized as biomarkers and valid prognostic factors of specific cancers. Although molecular mechanisms underlying dysregulated TRIM expression in cancer are not completely understood, there are general principles that account for tumor-related imbalances of TRIM proteins. Reduced expression levels of some tumor suppressive TRIMs can result from increased methylation of the corresponding gene promoters as described for *TRIM33* [40] and *TRIM58* [41]. The hypermethylation of CpG islands present in the promoter regions of the mentioned TRIM genes is pathologically relevant for chronic myelomonocytic leukemia (*TRIM33*) and lung cancer (*TRIM58*) patients. Alternatively, the low expression of the tumor-suppressive *TRIM8*, mainly observed in renal cell carcinoma but also in tissues from CRC patients (The Human protein Atlas, available from http://www.proteinatlas.org) and in the CRC cell line HCT116 [42], is inversely correlating with an overexpression of micro RNAs (miRs)-17-5p and miR-106-5p both of which are members of the miR-17-92 cluster. Importantly, the pathological overexpression of these *TRIM8* suppressing miRs, which is functionally relevant for increased chemo-resistance of several human cancers including CRC, is presumably caused by transcriptional induction by the proto-oncogene N-Myc [42]. Contrary to this, overexpression of some oncogenic TRIMs in various cancers is frequently due to the loss of miR dependent gene suppression as demonstrated—e.g., for *TRIM11* [43], *TRIM14* [44], *TRIM24* [45], *TRIM25* [46,47,48], and *TRIM44* [49,50]. However, since most miRs regulate several genes at once and thus can affect different pathways, the therapeutic supplement of these miRs may potentially induce various unwanted side effects.

**Figure 1:**
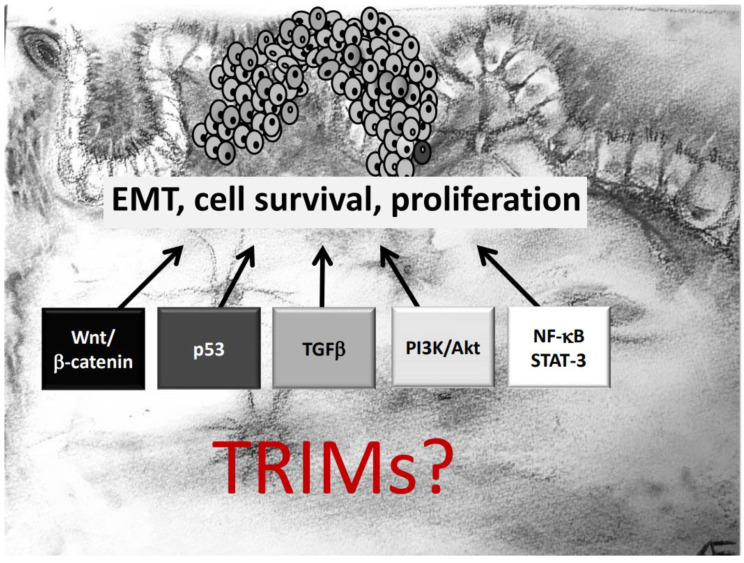
TRIM proteins can affect development and progression of colorectal cancer (CRC) via modulation of diverse oncogenic signaling pathways critically involved in epithelial–mesenchymal transition (EMT), survival, and proliferation of CRC cells.

Another important aspect which needs to be mentioned is that several TRIM genes are prone to chromosomal translocations, resulting in oncogenic gain-of-function fusion genes, which in turn induce a constitutive activation of oncogenic signaling pathways. Prominent examples include translocations of the *TRIM19* locus with the retinoic acid receptor α (RARα) gene, associated with acute promyelocytic leukemia [51]. Similarly, the BRAF gene is frequently translocated with *TRIM4* [52] or *TRIM24* in lung cancer [53] and melanoma [54]. This particular translocation results in the synthesis of the *TRIM24*-BRAF oncoprotein which impairs retinoic acid receptor signaling in a dominant negative manner thereby leading to aberrantly increased cell proliferation [21]. Moreover, chromosomal aberrations involving *TRIM24*, *TRIM27*, and *TRIM33* were observed in papillary thyroid carcinomas (PTC). An increasing number of publications have pinpointed the contribution of different TRIM proteins in tumor development with the majority of reports, demonstrating that TRIMs exert their diverse functional effects mainly through targeting specific signaling cascades relevant for tumorigenesis. Notably, depending on the tumor analyzed, one TRIM member can influence diverse mechanisms to influence biological processes like cell growth, migration, invasion, survival in an oncogenic fashion (Figure 1). In the next paragraphs we will give an overview of the main oncogenic signaling mechanisms directly or indirectly targeted by members of the TRIM family. For clarity, we will particularly highlight those reports of TRIM effects that are relevant for initiation and progression of CRC. A summary of TRIMs and their underlying mechanisms critically involved in CRC development is given in Table 1.

### 2.1. TRIMs and p53 Controlled Pathways

**Figure 2:**
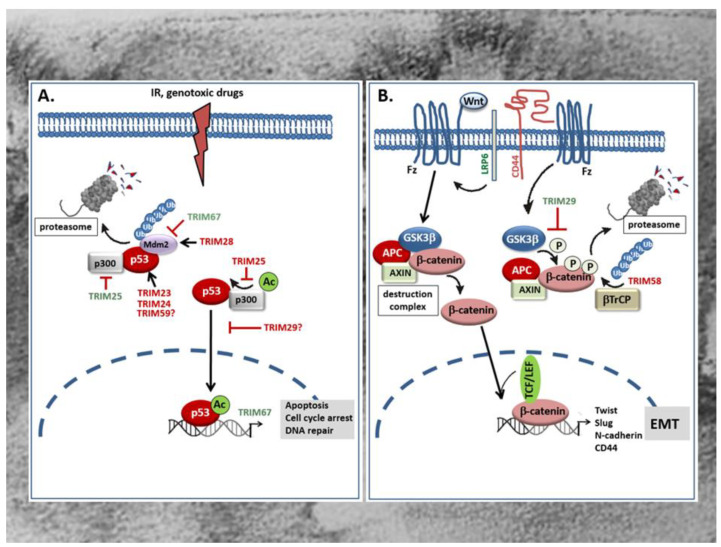
(**A**) Role of TRIMs in the control of p53 tumor-suppressor protein. TRIM proteins modulate the tumor suppressive activity of p53 e.g., in response to irradiation (IR) or genotoxic drugs via diverse mechanisms. P53 antagonizing TRIM proteins (red) can either directly interact with p53 and thereby promote p53 ubiquitination and proteasomal degradation or indirectly, via facilitating the association with the ubiquitin ligase Mdm2 which targets p53 for proteasomal degradation. Alternatively, the p53 negative regulatory TRIM25 interferes with the transactivation potential of p53 and consequently with the transcription of p53 controlled genes due to an impaired formation of a ternary p53 activation complex of p53, Mdm2, and p300 which is critical for p53 acetylation (Ac). Instead, the p53 inhibitory TRIM29 upon p300 dependent acetylation binds to p53 and enhances the sequestration of p53 in the cytoplasm thereby preventing p53-triggered transcription of p53 target genes relevant for tumor suppression. By contrast, some TRIMs promote stabilization of p53 either though directly binding to Mdm2 thereby preventing p53 proteasomal degradation (TRIM67) or by preventing the formation of a ternary complex of p53, Mdm2, and p300 which is also critical for p53 degradation as depicted for TRIM25. IR: irradiation; Mdm2: mouse double minute 2. (**B**) Opposing modulatory effects of TRIMs on Wnt/β-catenin signaling in the GI tract. TRIM29 induces Wnt/β-catenin signaling mainly through inhibition of β-catenin phosphorylation by GSK3β thereby preventing β-catenin degradation by the proteasome. Consequently, β-catenin can translocate into the nucleus and in a complex with TCF or LEF activate TCF/β-catenin-responsive genes including mesenchymal marker genes of EMT such as N-cadherin, Twist, Slug, and CD44. The increase in CD44 through its physical interaction with LRP6 can further enhance the Wnt-induced signaling processes. In contrast, TRIM58 inhibits β-catenin-dependent signaling by increasing β-catenin ubiquitination and proteasome-mediated degradation. TRIMs with a stimulatory effect on the Wnt/β-catenin pathway are marked in green; TRIMs with an inhibitory effect are red labelled. APC: Adenomatous polyposis coli; β-TrCP: β-transducin repeat-containing protein; Fz: frizzeled; GSK3: glycogen synthase kinase 3; LRP: lipoprotein receptor-related protein; TCF/LEF T-cell transcription factor/lymphoid enhancer factor.

The tumor suppressor protein p53 is mainly involved in the regulation of cell cycle, apoptosis, and the maintenance of genome stability [82]. Accordingly, mutations in the p53 gene leading to the expression of an inactive p53 protein are frequently found in human tumors and in more than 50% of all CRC patients [83,84]. Besides, an increased degradation or inactivation of functional p53 is found in tumors that have retained wild-type p53. Since p53 induces cell cycle arrest and apoptosis in response to irreparable DNA damage, the loss of p53 or impaired p53 activity is frequently associated with therapy resistance [84]. For this reason, recovery of wild-type p53 or reactivation of inactive p53 is supposed to be a promising approach for novel anticancer therapies [23]. Mechanistically, degradation of p53 by the proteasome is achieved through ubiquitination, mainly by the dimeric RING-type E3 ligase Mdm2 (Figure 2A). Consistently, overexpression of Mdm2 is frequently observed in early CRC and concomitant with a low p53 abundance [85]. Alternately to Mdm2, a regulation of p53 by TRIMs has meanwhile been observed in several human malignancies including CRC [86,87]. Without doubt, the regulation of p53 stability and/or activity reported for many TRIM proteins seems to represent the most eminent mechanism through which TRIM can modulate resistance of cancer cells towards chemotherapeutic drugs [87]. For an up-to-date overview of the role of p53 regulation by TRIMs in tumor chemotherapeutic drug resistance and tumor biology, we refer to reviews by our colleagues [86,87]. The majority of tumor promoting TRIMs belong to the p53 negative regulatory TRIMs either through an increased polyubiquitination and subsequent proteasomal degradation or, alternatively, by hindering p53 from entering the nucleus and thereby impairing its transcriptional activity. P53 inhibitory TRIM members which are characteristically overexpressed in CRC include *TRIM23* [58], *TRIM24* [59], *TRIM25* [62], *TRIM28* [67,69], and *TRIM29* [70]. *TRIM23* is significantly overexpressed in CRC, a characteristic associated with poor patient survival. Gene set enrichment analysis (GSEA) furthermore revealed that p53 and cell cycle signaling pathways related genes were enriched in patients with high *TRIM23* levels [58]. Mechanistically, *TRIM23* promotes proliferation of tumor cells mainly through an augmentation in p53 ubiquitination. Accordingly, a direct physical interaction of *TRIM23* with p53 could be demonstrated in HCT116 cells (Figure 2A) [58].

Similar to *TRIM23*, the overexpression of *TRIM24* which is synonymously known as transcription intermediary factor 1α (TIF1α), positively correlates with tumor size and shorter survival time of CRC patients as indicated by Kaplan–Meier survival analysis [59]. *TRIM24* represents a prominent member of negative regulatory TRIMs which can promote degradation of p53 via ubiquitination in a way independent of the master regulator of p53 stability, Mdm2 [60]. Since increased *TRIM24* levels observed in gastric cancer patients are functionally related to chemotherapy resistance [61], it is most likely that *TRIM24* constitutes an important oncogene in the development of gastrointestinal tumors preventing apoptosis in particular through the inhibition of p53. Similar to *TRIM24*, an increased expression of *TRIM59* has been demonstrated in CRC samples and is associated with advanced tumor stage of CRC patients [78,79]. Again, the role of p53 inhibition in the tumorigenic effects by *TRIM59* were not specifically investigated in colon carcinoma cells but data from gastric cancer demonstrated that *TRIM59* can physically interact with p53, thereby promoting p53 ubiquitination and degradation by the proteasome (Figure 2A) [88]. However, the finding that the transient knockdown of *TRIM59* had similar effects in p53 wild-type (HCT-116) and p53 mutated (SW480) cells is inferring that the tumor prevention upon *TRIM59* knockdown is p53 independent [78]. A further RING containing TRIM member with a mainly negative impact on p53 function in colon cancer is the estrogen responsive *TRIM25*. Importantly, this TRIM member was demonstrated to regulate p53 levels in colorectal cell line HCT116 through two distinct mechanisms [62]. Paradoxically, *TRIM25* inhibits the p53-triggered apoptosis although it increases p53 stability by preventing its ubiquitination and degradation by the 26S proteasome. Mechanistically, the inhibition of p53 degradation is due to the impaired formation of a ternary complex of p53, Mdm2, and p300 which itself is essential for polyubiquitination (Figure 2A). Apart from this, the same complex is required for acetylation of p53 and its transactivation potential to induce transcription of p53- controlled genes. Acetylation represents a critical posttranslational modification of p53 which facilitates the recruitment of transcriptional coactivators—e.g., p300—to the promoters of p53 responsive genes [89]. Thus, TRIM25 inhibits pro-apoptotic cell signaling induced by DNA damage mainly through blocking the transcriptional activity of p53 (Figure 2A) [62]. These data underscore the overall importance of *TRIM25* in connecting the ubiquitin proteasome system with different mRNA functions as previously reported for the control of metastatic gene signatures by *TRIM25* in breast cancer cells [90]. Conversely to the mentioned TRIMs, *TRIM28*—also known as transcription intermediary factor 1β (TIF1β) or KRAB-associated protein 1 (KAP1)—was identified as a direct Mdm2 binding protein which in a complex with Mdm2 promotes survival of HCT116 cells by targeting p53 for degradation (Figure 2A) [68]. Structurally, the interaction of *TRIM28* with Mdm2 is mediated by the CC domain of the TRIM protein. The formation of a ternary complex composed of *TRIM28*, Mdm2, and p53 is furthermore facilitated by MAGE (melanoma antigen) proteins which enhance the ubiquitin ligase activity of RING domain proteins including *TRIM28*. Interestingly, an increased abundance of MAGE proteins has been observed in various cancers [91]. Accordingly, a significant elevation in *TRIM28* levels has been demonstrated in different cancer tissues including those from CRC patients [67]. Clinically, increased expression of *TRIM28*—especially in stromal cells—is a marker of disease relapse and worse overall patient survival in CRC [69]. In addition to inhibition of p53, *TRIM28* can promote tumorigenesis through other mechanisms including its ability to act as a transcriptional co-repressor. Thereby, *TRIM28* in concert with Mdm2 promotes the formation of a p53 complex with histone deacetylase 1 (HDAC1) thus preventing acetylation of p53 [92]. However, the impact of this particular mechanism for CRC development is not known. For an overview of pleiotropic roles of *TRIM28* in cancer we refer to a previously published review by our colleagues [93].

A further member of TRIM family proteins with a clear tumorigenic role in CRC is *TRIM29* [70,71] also known as “AT group D complementing” (ATDC) gene product originally identified as a gene with a pathogenic role in the autosomal-recessive disorder Ataxia telangiectasia [72]. In particular, *TRIM29* is denoted as a marker of highly aggressive tumors and accordingly, upregulation of *TRIM29* predicts poor survival in CRC [94]. Notably, *TRIM29* belongs to the group of p53 inhibiting TRIMs although, due to the lack of a typical RING domain, it does not directly target p53 for degradation. Instead, *TRIM29* binds to p53 upon p300 dependent acetylation of *TRIM29* at Lys116, resulting in the sequestration of p53 in the cytoplasm thereby preventing p53-mediated transcription of its target genes in the nucleus (Figure 2A) [95]. In addition, *TRIM29* can bind to TIP60, a transcriptional coactivator of p53 and thereby promote its degradation in the cytoplasm [96]. Conversely, the histone deacetylase 9 (HDAC9), by targeting *TRIM29* for deacetylation, antagonizes the negative regulation of p53 by *TRIM29* resulting in increased p53 activity and reduced cell survival [97]. However, it is currently not clear which of these mechanisms are relevant for CRC development in humans.

In a clear contrast to the mentioned p53 negative regulatory TRIM proteins, as far as we are aware of, *TRIM67* represents the only p53 stabilizing TRIM with a pathological relevance in CRC. According to its p53 stimulatory effect, *TRIM67* is a classical member of tumor suppressive TRIMs. In line with the positive regulation of p53, *TRIM67* is downregulated in many CRC patients [81]. The epigenetic silencing of *TRIM67* in CRC tissues is mainly due to a hypermethylation of the *TRIM67* promoter at two loci (cg21178978 and cg27504802) when compared to the low methylation state measured in adjacent normal tissues. The functional impact of promoter methylation in the transcriptional silencing of *TRIM67* is furthermore underscored by the efficacy of the demethylation agent 5-aza-2 deoxycytidine to restore downregulated *TRIM67* mRNA expression in colorectal cancer cells. Mechanistically, *TRIM67* protects p53 from Mdm2-dependent degradation through the direct interaction with the C-terminus of p53, thereby inhibiting p53 ubiquitination by Mdm2 (Figure 2A). The authors of this study could furthermore demonstrate that in normal colon epithelial cells, *TRIM67* is a direct transcriptional target of p53 signaling in response to DNA-damaging drugs [81]. Their data clearly imply that the *TRIM67*-p53 axis is normally controlled by a positive regulatory loop that amplifies p53-induced cell cycle arrest and apoptosis in response to DNA damage and other stressors. Besides p53 modulation, TRIM proteins are implicated in various other tumor-relevant signaling pathways. The modulation of these pathways by different TRIMs in CRC will be discussed in the following sections.

### 2.2. TRIMs and Wnt/β-Catenin Signaling Controlled Pathways in CRC

The Wnt signaling pathway plays a critical role in the control of cellular processes including proliferation, migration, and adhesion and is highly relevant for normal embryonic development and adult tissue homeostasis [98,99]. Basically, the Wnt-signaling cascade amalgamates two distinct pathways, the non-canonical β-catenin-independent pathway and the canonical pathway which involves β-catenin, a co-activator of the T-cell transcription factor (TCF)/lymphoid enhancer factor (LEF) transcription factors which together activate genes that ensure, for example, the maintenance of multi-potency of crypt stem cells [98,100]. Usually, Wnt ligands bind to a heterodimeric receptor complex consisting of a frizzled (Fz) and a single transmembrane lipoprotein receptor-related protein (LRP) 5 or 6 co-receptor leading to a dissociation of β-catenin from the destruction complex, thus allowing its trafficking to the nucleus. In contrast, under conditions of unbound Fz/LRP receptors, β-catenin is clustered to the destruction complex and phosphorylated by GSK3 promoting its ubiquitination and subsequent proteasomal degradation. Further constituents of the destruction complex include the tumor suppressor protein axin, APC, CK1, protein phosphatase 2A (PP2A), and the E3 ubiquitin ligase β-transducin repeat-containing protein (β-TrCP) (Figure 2). Besides its role in crypt stem cell functions, β-catenin is a strong inducer of genes that mediate epithelial-to-mesenchymal transition (EMT), a central driver of cancer progression and metastasis. Accordingly, aberrant Wnt/β-catenin signaling is widely implicated in many malignancies, and frequently observed in tumors of the gastrointestinal (GI) tract [100,101]. Experimental evidence shows that constitutive hyperactivation of the canonical Wnt/β-catenin pathways, frequently in combination with mutations in other growth regulatory genes, results in the aberrant growth of epithelial cells [98,102]. An increased nuclear accumulation of β-catenin which under conditions of inactive Wnt signaling remains in the cytoplasm, was observed in up to 80% of CRC and thus supports the pathophysiological relevance of constitutive activation of the Wnt/β-catenin signaling cascade [98]. Importantly, most of these cancers harbor loss-of-function mutations in the APC gene, a negative regulator of the Wnt/β-catenin pathway [103]. In addition to genetic alterations in APC, the loss of Wnt/β-catenin suppressive mechanisms play a causative role in CRC.

Up to now, only two TRIM members—*TRIM29* and *TRIM58* which both exert opposing effects on tumors of the GI tract—have been identified to act mainly through modulation of Wnt/β-catenin signaling [37,71,77]. *TRIM29*, a member of the group of RING-less TRIMs, acts as a tumor promoting factor in CRC as implicated by the fact that increased expression levels of *TRIM29* observed in CRC patient specimens positively correlates with lymph node metastasis. By elucidating the underlying mechanisms in human CRC lines, Sun and colleagues demonstrated that overexpression of *TRIM29* inhibits β-catenin phosphorylation at Ser33 /Thr41 by GSK3β which in turn leads to an increase in β-catenin level due to its impaired proteasomal degradation (Figure 2B) [71]. The increase in total β-catenin by *TRIM29* is accompanied by nuclear β-catenin accumulation and induction of a TCF/β-catenin-responsive genes including mesenchymal marker genes of EMT such as N-cadherin, Twist, and Slug [71]. Furthermore, the authors demonstrate that *TRIM29* induces the Wnt/β-catenin signaling pathway through upregulation of CD44 expression which itself can enhance phosphorylation of GSK3β at serine 9 thereby preventing β-catenin phosphorylation (Figure 2B) [104]. CD44 itself constitutes a multifunctional transmembrane adhesion protein that physically associates with LRP6 upon Wnt-induction and modulates LRP6 activation and membrane localization. In line with its Wnt activating properties, CD44 is positively linked to progression of various human tumors [105]. Since CD44 itself is transcriptionally induced by β-catenin/TCF, it represents an important positive feedback-regulator of the Wnt/β-catenin signaling pathway [106]. Accordingly, activation of canonical Wnt signaling by *TRIM29* is perpetuated through transcriptional induction of CD44.

In contrast to *TRIM29*, *TRIM58* represents a tumor suppressive member of the TRIM family which is characteristically downregulated in CRC tumors. Low expression of *TRIM58* correlates with poor survival of patients suffering from CRC and is considered as a potential prognostic marker [37]. Mechanistically, *TRIM58* overexpression in CRC cell lines strongly inhibited CRC cell invasion mainly by suppressing the expression of EMT and matrix metalloproteinase (MMP) genes [37]. Similar tumor suppressive effects mainly related to an inactivation of β-catenin by *TRIM58* have been demonstrated in gastric cancer (GC) cell lines. Accordingly, *TRIM58* overexpression in GC cells caused a significant increase in β-catenin ubiquitination followed by its proteasomal degradation (Figure 2B) [77]. Collectively, these studies implicate that *TRIM58* exerts tumorsuppressive activities in the GI tract mainly through inactivation of Wnt/β-catenin signaling.

### 2.3. Involvement of TRIMs in TGFβ Signaling in CRC

**Figure 3:**
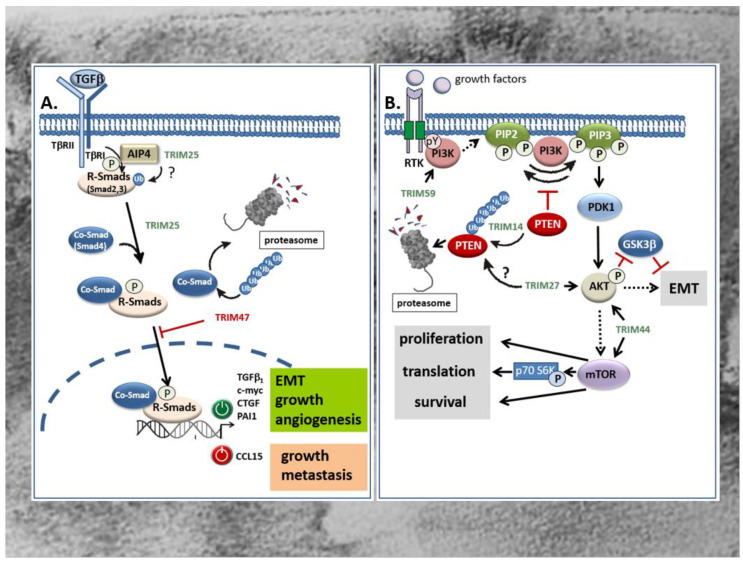
(**A**) Opposing modulatory effects of TRIM25 and TRIM47 on TGFβ-Smad signaling in the GI tract. TRIM25 induces the TGFβ signaling pathway as indicated from the increased phospho- Smad2 and Smad4 levels. Thereby, the activated Co-Smad/R-Smad complex transclocates into the nucleus and modulates transcription of target genes either in a positive (green symbol) or in a negative manner (red symbol). Whether in CRC TRIM25 may additionally promote the activation of R-Smad proteins by the TβRI through activation of Itch E3 ligase AIP4 through non-degradative ubiquitin modification remains questionable (question mark). In clear contrast, TRIM47 negatively interferes with TGFβ-Smad-signaling mainly by increasing ubiquitination and degradation of the Co-Smad protein Smad4. TRIMs with a stimulatory effect on the TGFβ-Smad signaling pathway are marked in green; TRIMs with an inhibitory effect are depicted in red. AIP4: Atrophin 1-interacting protein; CTGF: connective tissue growth factor; PAI: plasminogen activator inhibitor; R-Smads: receptor-Smads; TβR: TGFβ-receptor. (**B**) TRIM activation of PI3K/Akt signaling in CRC is executed by diverse mechanisms. TRIM59 promotes migration and invasion of CRC cells through direct activation of the PI3K/Akt pathway by an unknown mechanism, presumably through increasing tyrosine phosphorylation (pY) of the PI3k. TRIM14 enhances the PI3K-dependent phosphorylation of PIP2 to PIP3 indirectly through promoting the ubiqutination and subsequent proteasomal degradation of PTEN, an intrinsic antagonist of the PI3K. TRIM27 promotes proliferation and EMT of CRC cells mainly through increasing Akt phosphorylation. As a further consequence, phosphorylated Akt reduces β-catenin phosphorylation by GSK-3β resulting in an activation of EMT. Whether TRIM27 additionally facilitates ubiquitin-triggered degradation of PTEN is still questionable. TRIM44 activates the Akt/mTOR signaling pathway and its downstream target mTOR which, inter alia, results in the activation of the mitogenic p70 S6 kinase. TRIMs with a stimulatory effect on the PI3K/Akt signaling pathway are depicted in green. mTOR: mechanistic target of rapamycin; PIP2: phosphatidylinositol 4,5 bisphosphate (PIP2); PIP3: phosphatidylinositol 3,4,5 triphosphate; PDK1: phosphoinositide dependent protein kinase 1; PTEN: phosphatase and tensin homologue protein; RTK: receptor tyrosine kinase; p70S6K: p70 S6 kinase.

Members of the TGFβ superfamily are secreted cytokines crucially involved in the regulation of cellular processes including cell growth, differentiation, migration, autophagy, and apoptosis [107,108] Besides the prototypic TGFβ, other prominent members include the bone morphogenetic proteins (BMPs), activins, inhibins, and growth and differentiation factors (GDFs). Common to all members of the TGFβ superfamily, TGFβ signals through binding to type I and type II serine/threonine kinase receptors, thereby inducing a canonical signaling cascade via phosphorylation of receptor associated receptor (R)-Smad proteins which together with a common mediator of Smads (Co-Smads), namely Smad4, form transcriptionally active R-Smad/Smad4 complexes that translocate to the nucleus [108]. Most frequently, the binding of this complex to cognate promoter elements (Smad-binding elements) results in the transcriptional induction of TGFβ-controlled target genes [108]. Besides, diverse activities by TGFβ are exhibited by non-Smad pathways which either act independently of Smads, or cooperate with Smad signaling [109,110,111]. Prominent examples include the mitogen-activated protein (MAP) kinase-dependent pathway, the PI3 Kinase-triggered pathways, and the RhoA/Rock and Wnt signaling pathways [109,112]. According to the pleiotropic functions induced by TGFβ, perturbations of TGFβ signaling have been implicated in numerous human diseases including cancer [113]. Notably, with respect to tumor development and progression, TGFβ is a double-edged sword. During the early phases—e.g., by inducing cell-cycle arrest and apoptosis—TGFβ inhibits colorectal tumorigenesis [114,115]. However, in the late stages of tumor development, TGFβ promotes tumor progression by induction of proliferation, immunosuppression, angiogenesis, and EMT, the latter being mainly relevant for tumor metastasis [116,117]. Several studies revealed that members of TRIM proteins are critically implicated in the regulation of TGFβ signaling mainly through the targeted degradation of specific signaling modules (TGFβ-receptors, R-Smads, and Co-Smads). Along this route, different TRIM proteins have been demonstrated to modulate the canonical TGFβ-Smad signaling pathway both positively and negatively, depending on which TRIM member was involved [116,118]. To date, several TRIM family members including *TRIM11*, *TRIM25*, *TRIM26*, *TRIM28*, *TRIM33*, *TRIM47*, *TRIM59*, *TRIM62*, *TRIM66*, and *TRIM72* were shown to be directly associated with the TGFβ signaling pathway [118]. In the context of CRC, only *TRIM25* and *TRIM47* were shown to exert their tumorigenic features through a direct interference with TGFβ signaling (Figure 3A) [63,119]. Although both TRIM proteins were found to be significantly increased in colorectal tumors acting as tumor promoters, they affect TGFβ signaling in an opposite manner which underlines the ambivalent function of the TGFβ pathway in cancer. *TRIM25* which promotes development of CRC via different mechanisms induces proliferation, migration and invasion of HCT116 cells via induction of the TGFβ signaling pathway, as indicated by the increased level of TGFβ and BMPs. In addition, Sun et al. monitored a *TRIM25* dependent increase in Smad2 and Smad4 phosphorylation as a clear readout for activation of the canonical TGFβ-Smad pathway [63]. The functional impact of TGFβ receptor signaling on *TRIM25*-induced HCT116 cell proliferation was confirmed by the inhibitory effect of SB431542, a selective inhibitor of the TGFβ type I receptor (Alk5) (Figure 3A) [63]. With regard to the fact that the human promoter of the TGFβ_1_ gene itself is a target of autoregulation, the increase in Smad phosphorylation does not necessarily reflect a downstream event of TGFβ, but instead could be the reason for elevated TGFβ levels by *TRIM25*. In addition to the negative Smad regulation by ubiquitin modifications [116], non-degradative ubiquitin modifications can promote the activation state of R-Smad proteins as demonstrated for the Itch E3 ligase Atrophin 1-interacting protein (AIP4) [120]. From this point of view, it seems feasible that *TRIM25* may ubiquitinate Smad2 in a similar manner and thus promote its phosphorylation by the TGFβ receptor (Figure 3A). Further experimental data is needed to unravel the precise mechanisms underlying Smad activation by *TRIM25*. In contrast to *TRIM25*, *TRIM47* induces CRC proliferation through negatively interfering with TGFβ-Smad-signaling by increasing ubiquitination and degradation of the Co-Smad protein Smad4 (Figure 3A) [119]. The authors showed that loss of Smad4 caused an upregulation of the C-C motif chemokine ligand 15 (CCL15) which induced growth and invasion of human CRC cells via chemokine receptor 1 (CCR1) mediated signaling [119]. Importantly, CCL15 expression is blunted by TGFβ-Smad4 signaling which is functionally relevant for inhibition of colorectal cancer cell proliferation and metastasis at the early stages of CRC (Figure 3A).

### 2.4. TRIMs Affecting PI3K/Akt Signaling in CRC

Another intracellular signaling pathway with a high impact on carcinogenesis is the phosphoinositide-3-kinase (PI3K)/Akt pathway. This pathway, by virtue of transmission of extracellular growth factor-derived signals, has gained attention for cancer treatment since its dysregulation is frequently observed in most human malignancies including CRC [121]. In addition to somatic mutations in genes coding for one of three classes of PI3K (I-III) lipid kinases, the constitutive activation of PI3K in CRC can be due to increased activities of tyrosine kinases, e.g., the epithelial-derived growth factor (EGF) receptor or platelet-derived growth factor (PDGF) receptors, or other growth factors which also play a crucial role in CRC development. PI3K phosphorylates phosphatidylinositol 4,5-bisphosphate (PIP2) to yield phosphatidylinositol 3,4,5-triphosphate (PIP3) and synthesis of this lipids leads to the recruitment of the serine/threonine kinase Akt, synonymously called protein kinase B (PKB), the downstream target of PI3K (Figure 3B). Phosphorylation of Akt by the phosphoinositide dependent protein kinase 1 (PDK1) functionally associated with tumor growth and inhibition of apoptosis is also relevant for EMT (Figure 3B) [13,122]. The PI3K pathway is antagonized by intrinsic inhibitors including the lipid phosphatase and tensin homologue protein (PTEN) which dephosphorylates PIP3 to PIP2 (Figure 3B). Accordingly, a deficiency of this tumor suppressor results in the activation of the PI3K/Akt pathway as frequently observed in various cancers [123,124]. In addition, an increased activation of PI3K/Akt in CRC can rely on increased expression levels of some TRIM proteins as particularly demonstrated for *TRIM14* [56], *TRIM27* [66], *TRIM44* [75], and *TRIM59* [80]. Commonly, these TRIMs are considered potential biomarkers of CRC, since their upregulation in tissues of CRC patients correlated with a poor prognosis and an increase in some characteristic tumor features including invasion, metastasis, and apoptosis resistance. In the case of *TRIM14*, the increase in PI3K/Akt signaling indicated by elevations in Akt phosphorylation is mainly due to an increase in cytoplasmic colocalization of *TRIM14* with PTEN leading to an increased polyubiquitination and degradation of the intrinsic PI3K antagonist (Figure 3B) [56]. In a similar way, upregulation of *TRIM14* via induction of Akt signaling promotes migration and invasion and EMT progression of gastric cancer (Figure 3B) [44]. Accordingly, clinical data disclosed that the high *TRIM14* abundance frequently correlated with malignant features and unfavorable prognosis and was furthermore associated with lymph node metastasis and an advanced TNM stage [44]. Interestingly, the authors identified *TRIM14* as a direct target of miR-195-5p in GC and confirmed an inverse correlation between *TRIM14* mRNA and miR-195-5p in gastric cancer tissues. Whether a similar loss of miR-195-5p dependent *TRIM14* repression could also account for the upregulation of *TRIM14* in CRC is still elusive.

The relevance of TRIM-regulated PI3K/Akt pathway in CRC progression is further illustrated by *TRIM27*. Again, upregulation of *TRIM27* in CRC tissues correlated with several clinically relevant features including advanced tumor stage, increased lymph node metastasis and poor prognosis in patients diagnosed with CRC [66]. Following *TRIM27* depletion and overexpression in CRC cell lines, the authors of this study further demonstrated that *TRIM27* promoted cell proliferation and EMT mainly through increased Akt phosphorylation (Figure 3B) [66]. As a direct consequence, phosphorylated Akt reduces β-catenin phosphorylation at (Ser33/Thr41) by GSK-3β, resulting in an impaired β-catenin degradation [71]. However, the exact mechanism of *TRIM27*-mediated Akt phosphorylation in CRC is not known. Whether *TRIM27* mainly acts via interaction and ubiquitin-triggered degradation of PTEN, similarly as described for esophageal squamous cell carcinoma [125], needs to be elucidated by future investigations. In the case of *TRIM44*, induction of CRC cell proliferation, migration, and invasion by *TRIM44* seems mainly attributed to the increased activation of the Akt/mTOR signaling pathway (Figure 3B). First, GSEA on signal pathways by using the Kyoto Encyclopedia of Genes and Genomes database implicated that *TRIM44* was mainly enriched in the Akt/mTOR signaling pathway. In accordance with the results from bioinformatic predictions, expression levels of phosphorylated mTOR, Akt, and p70 were clearly attenuated upon siRNA-mediated silencing of *TRIM44* in the CRC cell line LOVO (Figure 3B) [75]. Importantly, mTOR is a common downstream target of Akt/PKB signaling and has emerged as an important promoter of cell growth/proliferation, protein translation, and angiogenesis in response to diverse growth factors, cytokines, and ligands of the toll like receptors (TLRs) mainly through activation of upstream protein kinases, namely PI3K and ERK [126]. In addition to the mentioned TRIM members, *TRIM59*, also denoted as mouse ring finger 1, is an oncogenic TRIM that is implicated in the pathogenesis of CRC at least partially via activating the PI3K/Akt pathway (Figure 3B) [80]. The authors of the study demonstrated that the expression of *TRIM59* was conspicuously overexpressed in CRC tissues and CRC cell lines and significantly correlated with TNM stage, lymph node metastasis, and with lower survival time of CRC patients [80]. As mentioned before, the significance of high *TRIM59* expression levels for prediction of poor prognosis was summarized by systematic review and meta-analysis by Wang and colleagues [79]. By investigating the underlying mechanisms of *TRIM59*, Sun et al. showed that knockdown of *TRIM59* suppresses the activation of the PI3K/Akt pathway in CRC cell lines as indicated by the strongly reduced levels of phosphorylated PI3K and Akt levels [80]. Conversely, the increased migration and invasion of the normal colon epithelial cells upon overexpression of *TRIM59* was prevented by a pharmacological inhibitor of PI3K (LY294002), thus supporting the functional impact of the PI3K/Akt pathway in the promotion of CRC cell migration and invasion and its regulation by *TRIM59* (Figure 3B) [80].

### 2.5. TRIMs Affect Proinflammatory Signaling by STAT3 and NF-κB

**Figure 4:**
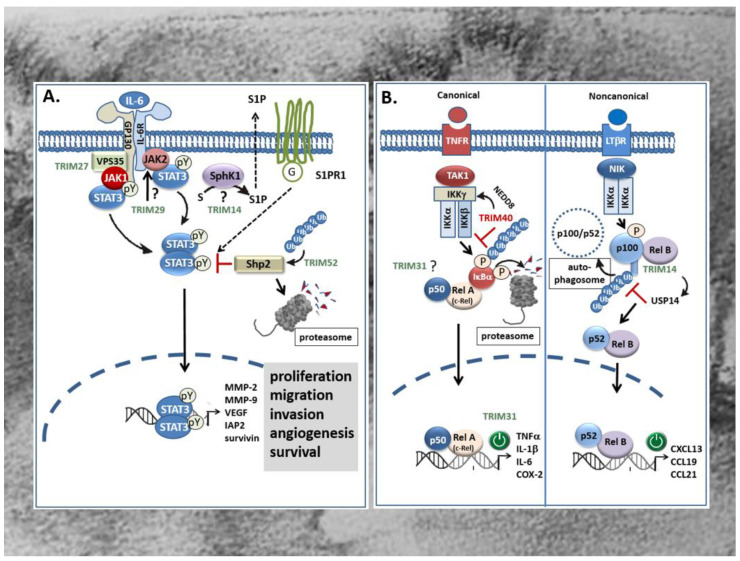
Effects of TRIMs on proinflammatory STAT3 (**A**) and NF-κB (**B**) signaling cascades in CRC. (**A**) TRIM14 promotes colorectal cancer cell migration and invasion mainly through induction of SPHK1 which by increasing the synthesis of S1P promotes activation of STAT3 and induction of STAT3-repsonsive genes through extracellular binding to the type-1 S1P receptor. However, the detailed mechanism of SPHK1 activation by TRIM14 is still unknown. TRIM27 can induce formation of JAK1/STAT3 containing complexes through physically interacting with the retromer complex VPS35 thereby facilitating STAT3 phosphorylation at tyrosine (pY). Instead, TRIM29 may enhance the constitutively active JAK2/STAT3 pathway presumably upstream of JAK2/STAT3 (question mark). By contrast, TRIM52 activates STAT3 signaling indirectly via promoting polyubiquitination and proteasomal degradation of Shp2, a protein tyrosine phosphatase involved in the negative regulation of STAT3. TRIMs with a stimulatory effect on STAT signaling are marked in green. G: G-protein; IAP2: inhibitor of apoptosis protein 2; JAK: Janus kinase; MMP: matrix metalloproteinase; SPHK1: human sphingosine kinase 1; S1P: sphingosine 1-phosphate; S1PR: S1P receptor; VEGF: vascular endothelial derived growth factor. (**B**) Overexpression of TRIM31 in CRC promotes invasion and metastasis of CRC cells through an activation of the canonical NF-κB pathway which leads to an upregulation of pro-inflammatory cytokines. By contrast, TRIM40 inhibits NF-κB mainly through promoting neddylation of IKKγ (synonym: NEMO) resulting in the stabilization of the IκBα protein and subsequently to the induction of NF-κB-responsive gene promoters e.g., those of diverse chemokines encoding genes. TRIM14 can activate the non-canonical NF-κB pathway and induction of RelB/p52 target genes by preventing p100/p52 from the autophagy-mediated degradation. Mechanistically, the direct physical interaction of TRIM14 with p100/p52 induces the recruitment of the deubiquitinating enzyme USP14 which cleaves K63-linked ubiquitin chains of p100/p52 preventing its autophagic degradation. TRIMs with a stimulatory effect on NF-κB signaling pathways are marked in green; TRIMs with an inhibitory effect are depicted in red. COX-2: cyclooxygenase-2; IKK: inhibitor of NF-κB kinase subunit γ; IL: interleukin; LTβR: Lymphotoxin β-receptor; NEMO: NF-κB essential modulator; NIK: NF-κB inducing kinase; TAK1: TGFβ activated kinase 1; TNFα: tumor necrosis factor α; TNFR: TNF-receptor; USP14: ubiquitin-specific protease 14.

As mentioned before, a large fraction of CRC tumors including sporadic CRC as well as colitis-associated cancer (CAC) display a constitutive activation of transcription factors known as prominent inducers of proinflammatory gene signatures. This includes some of the best characterized transcription factors like STAT3 [127,128] and members of the NF-κB protein family [14,129,130]. Increasing evidence arising from clinical and experimental data implicates a decisive role of these transcription factors in linking chronic inflammation to cancer development. Accordingly, both transcription factors are particularly relevant for development and progression of CAC but also for sporadic CRC [127,131]. Originally, the STAT signaling pathway was identified as a prominent interferon-inducible transcriptional pathway but meanwhile, it has been shown to control a wide variety of cellular processes, including cell growth, proliferation, angiogenesis, and immune responses [128,132]. The STAT3 signaling pathway is most potently activated by interleukin-6 (IL-6), but is additionally induced by other cytokines and growth factors including IL-10, IL-11, IL-22, EGF, PDGF, hepatocyte growth factor (HGF), as well as oncogenic tyrosine kinases (Figure 4A) [128,132]. These ligands upon binding to their respective receptors activate the Janus family kinases (JAKs) which in the case of cytokine receptors via direct binding to the linker protein gp130, induce the recruitment and subsequent phosphorylation of cytoplasmic STAT3 at tyrosine 705, in turn triggering its dimerization (Figure 4). Subsequently, dimerized STAT3 proteins translocate to the nucleus and induce the transcription of genes that promote proliferation, migration, invasion, angiogenesis, and cell survival (Figure 4A) [133]. In addition, activation of STAT3 in concert with other prominent transcription factors of inflammatory gene signatures such as NF-κB and AP-1 can enhance the expression of cytokines and chemokines particularly needed for recruitment of immune cells, thereby reinforcing the inflammatory STAT signaling [14]. Clinically, the constitutive activation, especially of STAT3 has been observed in more than 50% of colorectal tumors. However, since no activating mutations were identified so far, it is generally believed that the aberrant STAT3 signaling is mainly due to persisting signaling events caused by the dysregulation of specific signaling modules [14]. Notably, excessive TRIM-mediated STAT3 activation in CRC has been reported for several TRIMs, including *TRIM14* [55], *TRIM27* [65], *TRIM29* [70], and *TRIM52* [76]. Accordingly, induction of tumorigenic features in human CRC lines caused by induction of the STAT3 pathway is accompanied by increased expression levels of the respective TRIM protein in CRC tissues when compared to the matched non-cancerous tissues and associated with overall poor survival of patients. This clearly indicates that aberrant TRIM expression is clinically relevant for constitutive STAT signaling in CRC. *TRIM14* promotes colorectal cancer cell migration and invasion, mainly through induction of sphingosine kinase 1 (SPHK1) which by increasing the synthesis of sphingosine 1-phosphate (S1P) promotes activation of STAT3 in CAC [134] as well as in sporadic CRC (Figure 4A) [55]. Importantly, S1P is considered as a link between chronic inflammation and colon cancer [135]. Overexpression of *TRIM14* in CRC cells via increased STAT3 phosphorylation induced the expression of tumor relevant target genes of STAT3 including MMP-2, MMP-9 and vascular endothelial derived growth factor (VEGF) (Figure 4A) [55]. The precise mechanism through which *TRIM14* induces expression of SPHK1 in CRC [136] and particularly, whether ubiquitination is required for SPHK1 induction awaits further experimental evaluation.

In contrast to *TRIM14*, *TRIM27* can directly interfere with IL-6-induced STAT3 signaling events and downstream gene expression in CAC [65]. Overexpression of human *TRIM27* in nude mice induces the formation of JAK1-STAT3 containing complexes required for full phosphorylation of STAT3 upon IL-6 stimulation [65]. Intriguingly, confocal microscopy together with coimmunoprecipitation experiments revealed that IL-6 stimulation induces colocalization and physical interaction of *TRIM27* with the retromer complex VPS35, a large multimeric complex relevant for the retrograde transport of proteins from endosomes to the Golgi apparatus. VPS35 together with gp130, JAK1, and STAT3 build up the STAT3 activation complex (Figurer 4A). Hereby, the retromer, by acting as a platform for *TRIM27*-mediated assembly of the STAT3 activation complex, is critical for STAT3 phosphorylation [65]. Functionally, the deficiency of *TRIM27* in hematopoietic cells caused an attenuation of dextran sodium sulfate (DSS)-induced colitis in mice. In summary, this paper highlights the potential value of *TRIM27* as a novel therapeutic target particularly for treatment of inflammation-associated cancer [65]. Similar to *TRIM27*, *TRIM29* which again is upregulated in CRC tissues was found to exert oncogenic effects in CRC cells mainly through an activation of the JAK/STAT3 signaling pathway [70]. In terms of functional consequences, the knockdown of *TRIM29* significantly inhibited proliferation, migration, and invasion of CRC cells mainly through inhibition of prominent target genes of STAT3 critically involved in the regulation of the mentioned cell functions. The study by Xu and colleagues furthermore demonstrates that silencing of *TRIM29* suppressed the constitutively active JAK2/STAT3 pathway (Figure 4A) as indicated by the significantly decreased amounts of phosphorylated JAK2 and STAT3 proteins compared to cells transfected with control siRNA [70]. The results of the study further implicate that *TRIM29* activates STAT3 signaling upstream of JAK/STAT3 and thereby exerts diverse tumorigenic functions in CRC cells. A possible mechanism for STAT3 signaling pathway activation by TRIMs is exemplarily given by *TRIM52*. Overexpression of *TRIM52* enhanced polyubiquitination and proteasomal degradation of Shp2, one protein tyrosine phosphatase involved in the negative regulation of STAT3 (Figure 4A) [76]. Conversely, small hairpin (sh)RNA-mediated knockdown of *TRIM52* in CRC cells led to reduced phospho-STAT3 levels concomitant with an elevation in Shp2 expression [76]. Importantly, a similar correlation was also observed in tumors from xenograft mice, suggesting that *TRIM52* could act as an E3 ligase for Shp2 degradation also in vivo.

Another transcriptional activator factor that along with STAT3 plays a prominent role in CRC [14] and which is targeted by TRIMs is NF-κB, a family of dimeric transcription factors. Functionally, aberrant NF-κB activation demonstrated in the majority of colorectal and colitis-associated tumors, can follow two different routes: the canonical (synonymous: classical) route or the noncanonical (synonymous: alternative) activation pathway [137,138]. Activation of the classical route depends on the proteasomal degradation of inhibitor of NF-κB (IκBα) due to phosphorylation by the activated IκB kinase (IKK) complex leading to a release and subsequent activation of dimeric complexes of the NF-κB/Rel transcription family members p50, p65 (RelA) and c-Rel (Figure 4B) [14]. Accordingly, an increased binding of NF-κB has been demonstrated in 8 of 10 colorectal tumors [139]. Functionally, NF-κB promotes tumorigenesis primarily through induction of proinflammatory genes with high oncogenic potential such as cyclooxygenase-2 (COX-2), a prominent NF-κB target genes aberrantly overexpressed in CRC [14]. In contrast to the classical NF-κB pathway, the non-canonical NF–κB signaling mediates the activation of a complex of IKKα homodimer together with p52/RelB transcription factors (Figure 4B). Unlike the canonical pathway, this NF–κB route relies on the inducible phosphorylation–dependent ubiquitination and processing of the NF–κB precursor protein p100 by the action of the NF–κB-inducing kinase (NIK) (Figure 4B) [140]. For this alternative route of NF–κB signaling, up to now, only a few tumor activating ligands have been identified. Commonly, all of them constitute members of the tumor necrosis factor receptor (TNFR) superfamily including the lymphotoxin-β receptor, CD40, the B-cell activating factor receptor (BAFFR), and the receptor activator for NF–κB (RANK) [137,138]. Interestingly, depending on the TRIM analyzed, tumorigenic activities in CRC and CAC are achieved through activation of either of both NF–κB routes. Excessive *TRIM31* in CRC was found to promote invasion and metastasis of CRC cells through an activation of the canonical NF–κB pathway and is triggered by different pro-inflammatory cytokines [73]. Accordingly, overexpression of *TRIM31* in different CRC cell lines induced mRNA expression of TNF, IL-1β, and IL-6. Conversely, expression of these cytokines was significantly decreased upon shRNA-mediated knockdown of *TRIM31* [73]. The authors further demonstrated that *TRIM31* expression positively correlated with clinical staging in CRC patients and thus represents a valid indicator of poor survival. However, the mechanisms underlying *TRIM31* dependent cytokine expression were not further investigated.

In contrast, *TRIM14* was previously shown to activate the non-canonical NF–κB pathway by preventing p100/p52 from the autophagy adaptor protein p62-mediated autophagic degradation. Stabilization of the precursor protein p100 and its processing product p52 is achieved by *TRIM14* dependent recruitment of the deubiquitinating enzyme USP14 which cleaves K63-linked ubiquitin chains of p100/p52 and thereby destroys the signal tag for further autophagic degradation [57]. Structurally, *TRIM14* interacts with NF–κB p100/p52 through its PRY/SPRY domain which is highly relevant for mediating TRIMs interactions with RNA [29]. Thus, the interaction of *TRIM14* with USP14 promotes activation of noncanonical NF–κB signaling through interfering with autophagy. The broad functional impact of this regulation is implicated by the results from *TRIM14* knockout mice that exhibit impaired NF–κB triggered inflammation, as well as colitis and colitis-associated tumor development [57].

Contrary to these TRIM members, *TRIM40* represents an NF–κB inhibitory TRIM which is frequently downregulated in gastrointestinal cancers but highly abundant in normal gastrointestinal epithelia [74]. Mechanistically, *TRIM40* promotes neddylation of the inhibitor of NF–κB kinase subunit γ (IKKγ) also called NF–κB essential modulator (NEMO), a crucial regulator of canonical NF–κB activation (Figure 4B). Neddylation constitutes a specific posttranslational modification by the covalent tagging of the ubiquitin-like protein (neural precursor cell-expressed, developmentally downregulated gene 8) NEDD8 to a targeted protein. Consequently, the NF–κB inhibitor protein IκBα is stabilized and thus further impairs the activation of NF–κB, even in the presence of NF–κB activating cytokines [74]. Therefore, inhibition of NF–κB activity by *TRIM40* may constitute an important physiologic mechanism to prevent inflammation-associated carcinogenesis in the GI tract.

### 2.6. TRIMs Modulating Transcription and Translation in Colon Carcinoma Cells

Most of the aforementioned members of the TRIM family play complex and diverse roles in cancer biology at least partially arising from their multi-domain structure and their ability to build up homo- and hetero-di(multi)meric complexes [141]. Notably, a growing body of experimental evidence indicates that pleiotropic effects of many TRIMs in addition to their ubiquitin- and ubiquitin-like-modification capacity (sumoylation, neddylation, ISGylation) emerge from direct regulation of nucleic acids [142]. For instance, previous computational analysis predicted more than 15 TRIM proteins carrying RNA-binding properties and RNA-destabilizing activities although they contain no typical canonical RNA-binding domains [142]. Hereby, the requirement for E3 ligase activity in mRNA degradation is still unclear.

Using RNA affinity chromatography, we previously identified *TRIM25* as a novel caspase-2 mRNA-binding protein in human CRC cell lines, which reduces protein expression of caspase-2 mainly through interfering with caspase-2 translation [64]. Functionally, silencing of *TRIM25* caused a significant elevation in caspase-2 protein expression concomitant with an increased sensitivity towards intrinsic apoptosis of CRC cells induced by the topoisomerase inhibitors doxorubicin and etoposide [64]. Our data highlight the assigned role of caspase-2 to act as a damage sensor, driving cells into apoptosis at the last resort to genotoxic insults [143,144]. In addition to DNA damage-induced apoptosis, caspase-2 is essentially involved in the control of non-apoptotic functions such as genomic stability [145,146], cell-cycle checkpoint [147], tumor suppression [148], and autophagy [149]. Therefore, the *TRIM25*-mediated inhibition of these functions may be highly relevant for the malignant phenotype and increased therapy resistance of CRC cells. It is worth noting that, in a clear contrast to previous studies by Zhang et al. [62] who demonstrated that *TRIM25* dampened the p53-mediated DNA damage-induced apoptosis in the CRC cell line HCT116, the translation inhibitory effects by *TRIM25* were independent of the p53 status of the investigated CRC cell lines [64]. This implies that *TRIM25* may exert a broad anti-apoptotic program through diverse mechanisms. In addition to its RNA-binding capacity, the B-box domains of some TRIM proteins represent a hallmark zinc finger-DNA binding domain mainly relevant for transcriptional repressor activity. Again, this feature is exemplarily highlighted for *TRIM25* thus demonstrating the complexity of *TRIM25* contribution to cancer. By employing chromatin immunoprecipitation sequencing (ChIP-seq) and gene set enrichment analysis, *TRIM25* was identified as an important transcriptional regulator of breast cancer metastasis [90]. To the best of our knowledge, the relevance of *TRIM25* for tumor-related changes in the transcriptome of gastrointestinal epithelial cells has not been investigated so far.

Another TRIM member with a strong impact on transcription in CRC is *TRIM28*. Pathologically, several reports demonstrated the correlation between high *TRIM28* expression level and the worse overall patient survival and disease-recurrence mainly in epithelial cancers including CRC [69]. The contributions of *TRIM28* to cancer are complex and mediated by different mechanisms including the inhibition of p53 activity, the activation of DNA damage repair mechanisms, induction of EMT, and maintenance of stem cell pluripotency, just to cite a few examples [93]. Many of these pleiotropic properties of *TRIM28* are attributed to its role as a nuclear transcriptional co-repressor of a plethora of regulatory networks [93]. Thereby, a large number of transcription factors of the Krüppel-associated box (KRAB) repressor domain of zinc finger proteins (ZFPs) are thought to be directly targeted by *TRIM28.* This interaction triggers the recruitment of various heterochromatin inducing factors which finally lead to repression of transcription [150]. The relevance of gene repression by this particular family of repressor proteins is reflected in the large number of the KRAB zinc finger protein genes in the human genome [151]. However, accumulating evidence implies that the main role of *TRIM28* is presumably not mediated by the co-repression through KRAB-ZFPs [152]. Instead, *TRIM28* inducible effects on EMT do mainly result from activation of EMT relevant gene expression by *TRIM28* binding to specific promoter elements, the so called fibroblast transcription site-1 (FTS-1) [153]. This promoter element, originally identified in the promoter region of the fibroblast-specific protein 1 (FSP1) which is specifically upregulated during EMT and relevant for the characteristic phenotype of stromal fibroblasts [154]. Interestingly, the high aggressiveness of CRC tumors with high *TRIM28* expression levels is probably mediated by *TRIM28* dependent induction of tumor promoting signaling pathways in the tumor stroma and pro-survival pathways in tumor epithelial cells [67,69]. Collectively, these examples reinforce the functional impact of certain TRIM proteins in coordinating transcriptional and post-transcriptional gene regulation by the ubiquitin proteasome system.

## 3. Oncogenic features affected by TRIMs

### 3.1. TRIMs and (De)Regulation of Apoptosis and Autophagy

Clinically, the large majority of CRC patients (90%) classified to stage IV tumors die from disease progression mainly due to drug resistance [155]. The impaired sensitivity to 5’fluorouracil (5-FU), a drug commonly used as a first-line chemotherapeutic agent for adjuvant therapy of CRC is mainly attributed to defects in apoptosis programs. Hereby, evasion of apoptosis through the inactivation of p53 pathways essentially contributes to hallmarks of cancer including sustained cell proliferation, inappropriate cell cycle arrest, loss of genomic integrity, and therapy resistance in CRC [156]. Given the high prevalence of p53 deregulation in the pathogenesis of CRC, the regulatory impact of several TRIMs through their control of expression of p53 target genes which are particularly relevant for cell growth and apoptosis, seems immense. As described before, the majority of TRIMs functionally implied in the pathogenesis of CRC (e.g., *TRIM23*, *TRIM24*, *TRIM25*, *TRIM28*, and *TRIM29*) promote p53 degradation pathways either directly, or through increasing the activity of the E3 ligase Mdm2. Accordingly, ablation of these TRIMs, either through increasing the levels of p53, or via enhancing p53 activity in the nucleus leads to the induction of p53 target genes such as p21 thereby preventing cell cycle transition and reinforcing DNA damage-induced cell death. By contrast, inhibition of p53 degradation pathways triggered by a few members of the TRIM family (*TRIM67*, *TRIM8*) essentially contributes to their overall tumor suppressive features. Importantly, both of the p53 stabilizing TRIM proteins are direct transcriptional targets of p53 and under the positive control of p53 responsive elements in the respective gene promoters [81,157]. However, some TRIMs were shown to promote colon cancer independently of p53 signaling. Knockdown of *TRIM59* for example can inhibit malignant processes including evasion of apoptosis in p53 wild-type cells (HCT116) as well as in the p53 mutant CRC cell line SW480 [78]. These findings fit to our previous studies which demonstrated that knockdown of *TRIM25* via upregulation of the pro-apoptotic caspase-2 similarly sensitizes wild-type p53 (RKO) and p53 mutated (DLD-1) CRC cells to DNA-damage-induced apoptosis [64] thus inferring that some TRIMs exert anti-apoptotic programs which do not rely on the inhibition of p53. Although not particularly highlighted in CRC, many of the TRIMs previously described as modulators of other main oncogenic signaling pathways—including NF-κB, STAT3, Wnt/β-catenin, TGFβ, and PI3 kinase—may indirectly affect the outcome of apoptosis and consequently, the responses of colon carcinoma cells toward chemotherapeutic drugs (Figure 1). For instance, *TRIM31* can trigger chronic inflammation, invasion and metastasis in CRC mainly through activating the NF-κB pathway [73]. Coincidental to this finding, another report could demonstrate that inhibition of NF-κB increases the sensitivity of CRC cells to 5´fluorouracil [158]. Mechanistically, activated NF-κB directly contributes to drug resistance in CRC through induction of the multi drug resistance protein 1 (MDR1) gene expression [159]. An additional level of complexity is given by the crosstalk between different oncogenic signaling pathways in CRC (reviewed in [13]). Prominent examples include the intrinsic apoptosis inhibitors, Survivin and XIAPs, which are commonly induced by PI3K-Akt but also by the Wnt/β-catenin pathway and inhibited by the TGFβ-Smad pathway [160]. Another example is *TRIM24* which was shown to inhibit apoptosis of CRC cells mainly through increasing Bcl-2 and attenuating the expression of caspase-3 and PARP through mechanisms that still need to be unveiled [161]. Since *TRIM24* has also been described as a co-activator of STAT3 in glioblastoma, it cannot be excluded that a similar mechanism may also account for chemoresistance by *TRIM24* in CRC. Therefore, it is tempting to suggest that modulation of these pathways by certain TRIMs may additionally contribute to impaired apoptosis and to therapy resistance of CRC cells.

Autophagy, originally described as a major cellular recycling mechanism, is meanwhile considered as a pathway targeting selective substrates for degradation [162]. Similarly to apoptosis, autophagy constitutes a typical type of programmed cell death but the relationship between both pathways is diverse in tumor development ranging from a pure synergism to a strong antagonism with the latter being highly relevant for tumor cell survival, especially under stress conditions due to exposure of cells to chemotherapeutic agents [163]. The interaction between both types of programmed cell death in CRC is most prominently regulated by the PI3K/Akt/mTOR pathway [163]. Regulation of autophagy is thought as a functionally relevant feature shared by many TRIM members. Along with modulating p53 stability, TRIMs can impact on tumor progression through directly affecting autophagy [162,164,165]. Notably, the underlying mechanisms are complex and context-specific. Some TRIMs can affect autophagy indirectly either through modulating abundance of autophagy-related proteins transcriptionally through modulation of diverse transcription factors [57,166], or through repressing the expression of specific miRNAs that target some autophagy key factors [167,168]. Moreover, TRIMs can directly affect activity of autophagy regulators e.g., by acting themselves as autophagy receptors or by facilitating the assembly of constituents of the autophagosome such as Beclin-1 or the protein kinase ULK1 [164]. In terms of intestinal inflammation and cancer, a direct contribution of autophagy has been demonstrated for *TRIM11* [169] and *TRIM14* [57]. A previous study by Xie and colleagues reported on the identification of *TRIM11* as an E3 ubiquitin ligase which mediates polyubiquitination and subsequent autophagic degradation of the receptor interacting protein kinase 3 (RIPK3) as novel regulatory mechanisms of negative regulation of RIPK3 which is relevant for antagonizing necroptosis [169]. Functionally, hyperactivation of mTOR—e.g., as induced by western diet and dysbiosis—induces necroptosis of intestinal epithelial cells mainly through increasing RIPK3 abundance. From their data, it is tempting to suggest that *TRIM11* exerts a protective role in the gut mainly through antagonizing intestinal inflammation and cancer. In a clear contrast, *TRIM14* negatively interferes with the autophagic degradation of the NF-κB family member p100/p52 and thereby induces noncanonical NF-κB signaling pathway as described above (Figure 4B) [57].

### 3.2. TRIMs and EMT

Beyond doubt, the modulation of EMT accounts as a critical feature through which various TRIM proteins contribute to intestinal tumorigenesis. EMT represents a developmental process of transformation of epithelial cells to mesenchymal cells. During EMT, marker proteins of epithelial differentiation are characteristically downregulated whereas mesenchymal genes are transcriptionally induced. Since the increased abundance of mesenchymal markers is furthermore accompanied by the breakdown of epithelial cell–cell interactions and basement membrane mainly through the upregulation of MMPs, this process is closely associated with tumor invasion and early metastasis [170]. Furthermore, some previous reports have highlighted the significant role of some TRIM family members in EMT-triggered cancer stem cell (CSC) acquisition and renewal [171]. Due to their capability to differentiate and self-regenerate, CSC are endowed with a high metastatic potential and resistance to chemo- and radiotherapy [171]. Since EMT is controlled by different oncogenic signaling pathways, including Wnt/β-catenin, Notch, Hedgehog, TGFβ, and PI3K the TRIM dependent modulation of these pathways and related signaling modules is probably of significant relevance for EMT. However, since most of these signaling pathways are not linear but closely interconnected, decoding the specific impact of a TRIM controlled determinant for EMT is difficult. Members of the TRIM family explicitly reported to affect EMT in the context of CRC are *TRIM27*, *TRIM28*, *TRIM58*, and *TRIM59*.

*TRIM27*, a member of the class IV of TRIMs which commonly bear PRY and/or SPRY domains at their C-terminus, is a TRIM characteristically upregulated in CRC tissues. As mentioned before, the increased expression of this TRIM member directly associates with several clinicopathologic features, e.g., tumor stage, overall survival, metastasis, and relapse [66]. Functionally, siRNA-mediated knockdown of *TRIM27* caused an increase in E-cadherin expression concomitant with an attenuation of the mesenchymal marker proteins N-cadherin and vimentin [66] suggesting that *TRIM27* can induce EMT in CRC cells. However, the precise mechanisms of increased Akt phosphorylation observed upon overexpression of *TRIM27* warrants further investigation.

In the case of *TRIM29*, the direct tumor promoting effects by TRIMs on CRC could be clearly assigned to an induction of EMT [71]. Hence, a previous study has demonstrated that potential *TRIM29* target genes are functionally related to EMT relevant pathways thus suggesting that *TRIM29* may constitute a key regulator of EMT [172]. As already described in Section 2.2, *TRIM29* induces EMT through activation of the Wnt/β-catenin signaling pathway mainly via upregulation of CD44 [71]. CD44 itself can promote EMT in many cancer types mainly through induction of mesenchymal markers and downregulation of epithelial markers [173]. The functional impact of *TRIM59* in the induction of colorectal EMT processes is mainly reasoned by results from transient knockdown experiments. Thereby, Sun et al. demonstrated that depletion of *TRIM59* in CRC cell lines significantly reduced expression of vimentin and Snail while inducing the expression of E-cadherin implicating that downregulation of *TRIM59* prevents the progression of EMT mainly through modulating EMT-related gene expression [80]. The detailed mode of action how *TRIM59* differentially influences the expression of master regulators of EMT needs to be investigated further.

In contrast, reduced expression of the tumor suppressive *TRIM58* is thought to be an eligible marker for early CRC detection. *TRIM58* exerts a clear tumor suppressive activity in CRC mainly through its critical role in limiting Wnt/β-catenin dependent EMT [37]. Interestingly, ectopic expression of *TRIM58* in CRC cells exclusively interfered with tumor cell invasion mainly through inhibition of EMT and MMP regulation, without affecting other malignant properties, such as cell proliferation or migration [37]. Mechanistically, the *TRIM58*-induced inhibition of invasion was mainly achieved through the negative regulation of β-catenin and subsequent downregulation of Snail and Slug transcription factors which together lead to the stabilization of cell–cell adhesion and basement membrane integrity [37]. To answer the question whether the reduction in Wnt/β-catenin signaling does mainly result from *TRIM58*-dependent ubiquitination and degradation by the proteasome as reported for GC cells [77] requires further investigation.

## 4. Concluding Remarks

In this review, we summarized recent advances in deciphering the diverse roles of TRIM proteins in CRC development and progression. During the last years, an increasing number of studies has documented that TRIM proteins are being abundantly expressed in tissues from patients with CRC when compared to matched non-cancerous tissues. With respect to the finding that overexpression of most TRIM members tightly correlated with low overall patient survival rates and disease recurrence, TRIMs represent attractive therapeutic targets and novel tumor markers for early diagnosis and risk assessment as well as therapy of CRC. The prominent role of these cancer promoting TRIMs in CRC development is substantiated by data from loss-of-function and gain-of function in CRC cell lines and from experiments with xenograft models. In contrast, a few members including *TRIM8*, *TRIM15*, *TRIM24*, and *TRIM40* are characteristically downregulated in CRC and exert tumor-suppressive activities. Making things even more complicated, some TRIM proteins were shown to exhibit a dual role either as an oncogene or as tumor suppressor, depending on the tumor (cell)-type and context. Therefore, unveiling the crosstalk with other key functions of a particular TRIM is an absolute requirement before it can be assigned as a bona fide therapeutic target for treatment of CRC. A further level of complexity is given by the fact that mechanisms underlying tumor modulatory roles of a certain TRIM protein are diverse and may simultaneously impact distinct tumorigenic features including apoptosis, EMT, metastasis, therapy resistance, and inflammation. Hence, it is known that more than 50% of all TRIM proteins are involved in the control and orchestration of innate immunity which critically participates in the initiation and progression of CAC and CRC. Likewise, an estimation of therapeutic benefits promised by targeting a particular TRIM member remains difficult since a single TRIM member is able to influence different signaling processes. Therefore, the crosstalk of different signaling pathways targeted by TRIMs has to be considered as well (for review [174]). Previous studies furthermore demonstrated that several TRIM proteins, including *TRIM25* represent important hubs connecting the ubiquitin protein modification pathway directly with different RNA functions, especially mRNA stability and mRNA translation. Interestingly, several of these TRIMs contain typical zinc finger DNA-binding domain analogues to *TRIM25* implicating that these TRIMs, in addition to posttranslational protein modifications, can directly modulate gene expression at the transcriptional and/or post-transcriptional level [29,64,90]. Given the pleiotropic roles exhibited by TRIMs in the control of oncogenic signaling pathways and their frequent deregulation in human cancers, TRIMs represent attractive drug targets for novel anticancer therapies. Indeed, targeting of E3 ubiquitin ligases including Mdm2, XIAPs, or RNFs by either small molecular weight inhibitors or by neutralizing antibodies is currently under investigation with some of these inhibitors already reached clinical trials (for review [23]). Noteworthy, several of reported TRIM actions rely on ubiquitination-independent effects but depend on the direct binding to interacting partners including proteins and nucleic acids. A profound insight into structural aspects of TRIM interactions with these different targets—e.g., by analysis of crystal structures and 3D modeling—is an important prerequisite for rational drug design. Also, further investigations are needed for unraveling the exact role of individual TRIMs with respect to their different cellular context and possible tumor-stage activities during CRC development. In any case, such studies undoubtedly will provide therapeutically valuable insights and enrich our understanding of the pathogenesis of CRC.

## Figures and Tables

**Table 1:** Summary of TRIM family members with ascertained roles in the development and progression of tumors of the gastro intestinal tract (GIT) and their modulatory effects on oncogenic signaling pathways (↑: activation; ↓: inhibition). In addition, the table depicts the aberrant expression levels of respective TRIM members in CRC tissue in comparison with normal tissue. n.d.: not determined; TF: transcription factor.

TRIM	Role	Affected Pathway	Expression in Tumors	Reference
*TRIM11*	oncogenic	n.d.	increased	[43]
*TRIM14*	oncogenic	STAT3 (via SPHKK1) ↑	increased	[55]
	oncogenic	PI3K/AKT ↑	n.d.	[44,56]
	oncogenic	NF-κB (non-canonical) ↑		[57]
*TRIM23*	oncogenic	p53 degradation ↑	increased	[58]
*TRIM24*	oncogenic	p53 degradation ↑	increased	[59,60,61]
*TRIM25*	oncogenic	p53 activity ↓	increased	[62]
	oncogenic	TGFβ/Smad ↑	increased	[63]
	oncogenic	Caspase 2 translation ↓	n.d.	[64]
*TRIM27*	oncogenic	STAT3 ↑	increased	[65]
	oncogenic	PI3K/AKT ↑		[66]
*TRIM28*	oncogenic	p53 activity ↓	increased	[67,68]
	oncogenic	repression of TFs		[69]
*TRIM29*	oncogenic	STAT3 ↑	increased	[70]
	oncogenic	Wnt/β-catenin ↑	increased	[71]
	oncogenic	nuclear p53 translocation ↓?	increased	[72]
*TRIM31*	oncogenic	NF-κB (canonical) ↑	increased	[73]
*TRIM40*	tumorsuppressive	NF-κB (canonical) ↓	decreased	[74]
*TRIM44*	oncogenic	Akt/mTOR ↑	increased	[75]
*TRIM52*	oncogenic	STAT3 ↑	increased	[76]
*TRIM58*	tumorsuppressive	Wnt/β-catenin ↓	decreased	[37,77]
*TRIM59*	oncogenic	p53 degradation ↑?	increased	[78,79]
	oncogenic	PI3K/AKT ↑	increased	[80]
*TRIM67*	tumorsuppressive	p53 degradation ↓	decreased	[81]
